# The current clinical practice of general orthopaedic surgeons in the treatment of lateral ankle sprain: a questionnaire survey in Miyazaki, Japan

**DOI:** 10.1186/s12891-021-04527-8

**Published:** 2021-07-24

**Authors:** Takuji Yokoe, Takuya Tajima, Nami Yamaguchi, Yudai Morita, Etsuo Chosa

**Affiliations:** Division of Orthopaedic Surgery, Department of Medicine of Sensory and Motor Organs, Faculty of Medicine, University of Miyazaki, 5200 Kihara, Miyazaki, Kiyotake 889-1692 Japan

**Keywords:** Lateral ankle sprain, Clinical practice, Orthopaedic surgeons, Questionnaire

## Abstract

**Background:**

Lateral ankle sprain (LAS) is one of the most common musculoskeletal injuries. Numerous studies regarding LAS have been performed. However, there are few studies evaluating the current clinical practice of orthopaedic surgeons regarding LAS. The purpose of this study was to evaluate the current clinical practice of general orthopaedic surgeons in the treatment of LAS.

**Methods:**

A questionnaire survey was conducted from September 2020 to December 2020 in Miyazaki, Japan, to evaluate the clinical practice of general orthopaedic surgeons in the treatment of LAS. The survey was composed of 12 questions that were developed with consideration of the recommendations in the current clinical practice guidelines (CPGs) published by the Dutch orthopaedic society. The questions in this study were focused on the diagnosis, conservative treatment, rehabilitation, and the criteria for return to sports (RTS).

**Results:**

The survey response rate was 82.7% (129/156). Among the respondents, 95.3% did not consider the Ottawa Ankle Rules in the decision to perform plain radiography for patients. Rehabilitation following LAS was performed in 58.9% of patients. Eighty-five (65.9%) of the surgeons used only one factor as the criterion for RTS. The absence of pain was the most frequently used criterion (45.7%). No objective criteria were used for the RTS decision in athletes with LAS.

**Conclusions:**

The present study suggested that most general orthopaedic surgeons do not provide the care for patients with LAS recommended by the current CPGs. No objective criteria for the RTS decision are used for athletes with LAS.

## Background

Lateral ankle sprain (LAS) is one of the most common musculoskeletal injuries, with an incidence of 2.15 person-years in the United States [1]. Nearly half of ankle sprains were reported to occur during sports activities, with indoor sports, basketball, and soccer reported to be high-risk sports [1, 2]. If LAS is not properly treated, 20–74% of patients will result in chronic lateral ankle instability (CLAI) [3–5]. However, even if patients with LAS receive appropriate treatment, they can still develop CLAI [6], indicating the need for better management of LAS. Patients with a history of LAS and CLAI are at risk for the development of posttraumatic osteoarthritis of the ankle [7, 8]. According to the current clinical practice guidelines (CPGs), conservative treatment is the gold standard for LAS [9, 10]. In order to prevent negative results following LAS, a greater advance in the diagnosis, treatment, rehabilitation, and prevention of LAS is still needed.

Especially, the improvement of the clinical practice of emergency physicians and general orthopaedic surgeons may be critical because the majority of individuals who seek medical treatment for LAS visit an emergency department or private orthopaedic clinic [11–14]. Many previous studies have evaluated issues regarding the management of LAS in the emergency department, such as the validity of the Ottawa ankle rules (OARs) [14–17]. However, few studies have investigated the clinical practice of general orthopaedic surgeons in the management of LAS [13]. It remains unclear whether or not orthopaedic surgeons are proficient in the management for LAS. Kucera et al. reported that subjects with a history of ankle sprain had an approximately 3.5 times greater risk of recurrent injuries than those who had no history of ankle sprain [18]. Better management by primary physicians, including general orthopaedic surgeons, may prevent recurrent injuries in patients with LAS. We hypothesized that many general orthopaedic surgeons would not provide care for LAS in accordance with the recommendations of the current CPGs. In addition, there is a lack of evidence-based criteria for the return to sports (RTS) following conservative treatment for LAS [19–21]. A premature RTS after insufficient assessment may contribute to a high rate of reinjury and the development of CLAI [22, 23]. If evidence-based RTS criteria were applied to athletes with LAS, the high rate of reinjury and development of CLAI may be reduced. Thus, it is worth knowing what criteria are used in the decision-making in relation to the RTS for athletes with LAS in the clinical setting. The purpose of this study is to report the results of a questionnaire survey regarding the current clinical practice of general orthopaedic surgeons in the management of LAS.

## Methods

This study was approved by the institutional review board of our hospital. From September 2020 to December 2020, questionnaires were sent by post to the orthopaedic surgeons who worked in Miyazaki prefecture, Japan. Based on a list of orthopaedic surgeons in Miyazaki, those who had retired or did not see patients with LAS were excluded from this study. All questionnaires completed by the orthopaedic surgeons were included in this study. Miyazaki prefecture is a rural area located in the south of Kyushu in Japan; the population is almost 1,060,000. Most orthopaedic surgeons in Miyazaki see a broad spectrum of patients in the clinical settings because there are few specialized hospitals or institutes for specific orthopaedic diseases. Therefore, it was considered suitable for evaluating the clinical practice of general orthopedic surgeons in the management of LAS. In addition, the study population was selected with consideration of the accessibility of information regarding the doctors or their clinics and hospitals, as well as the contact information.

A questionnaire composed of a total of 12 questions, of which 11 were closed-ended questions (single answer), and one was an open-ended question (Table 1). There were 4 questions regarding the diagnosis of LAS, and other questions were about conservative treatment and rehabilitation for LAS. One question was related to athletes with LAS. The respondents were instructed to answer their best managements for patients with LAS who were treated conservatively. The questionnaires were developed by the authors to evaluate clinical practice of general orthopaedic doctors in the diagnosis, treatment, and rehabilitation for LAS. Evidence-based CPGs have been published by the Dutch Orthopaedic Society [9, 10]. The included questions were discussed and selected among the study members based on recommendations of these CPGs. Moreover, there is a lack of evidence-based criteria for a RTS after LAS [19–21]. Therefore, an open-ended question was used to investigate the criteria used by general orthopaedic surgeons in decision-making in relation to the RTS after LAS. The Cohen’s Kappa coefficient (κ) was calculated to assess the reliability of closed-ended questions using results from 30 randomly selected respondents. The respondents answered the questionnaire twice with a two-week interval. All values of κ were > 0.80, indicating almost perfect according to Landis’s classifications (slight, 0.0–0.20; fair, 0.21–0.40; moderate, 0.41–0.60; substantial, 0.61–0.80; almost perfect, 0.81–1.00) [24]. All collected questionnaires were tabulated by an author (T.Y.).

**Table 1:** The Questionnaire used in this study

1. The diagnosis of lateral ankle sprain (LAS)
Q.1 Do you perform plain radiography for the diagnosis of LAS ?
□ Yes □ No
Q.2 Do you consider the Ottawa Ankle Rules when deciding whether or not to perform plain radiography?
□ Yes □ No
Q.3 Do you perform stress plain radiography for the diagnosis of LAS?
□ No □ Only inversion stress □ Only anterior drawer □ Inversion stress and anterior drawer
Q.4 Do you perform ultrasonography for the diagnosis of LAS?
□ Yes □ No
2. Conservative treatment and rehabilitation for LAS
Q.5 Do you immobilize the injured ankle for the treatment of LAS?
□ Yes □ No
Q.6 If yes in Q.5, which do you prefer a splint or cast for immobilization of the injured ankle? (A cast means a hard cast created with plaster. A splint includes any type of brace, air cast, and walking boot.)
□ Splint □ Cast □ Splint or Cast
Q.7 If yes in Q.5, how long is the mean duration of immobilization of the injured ankle in the treatment of LAS?
□ < 1 week □ 1–2 weeks □ 2–3 weeks □ 3–4 weeks □ > 4 weeks
Q.8 Do you instruct a patient with LAS to avoid weight bearing on the injured ankle?
□ < 1 week □ 1–2 weeks □ 2–3 weeks □ 3–4 weeks □ > 4 weeks
Q.9 Do you instruct a patient with LAS to wear an ankle supporter after immobilization?
□ Yes □ No
Q.10 Do you consider a rehabilitation program for patients with LAS? If yes, how long is the mean duration of rehabilitation?
□ No □ < 2 weeks □ 2–4 weeks □ 4–6 weeks □ 6–8 weeks □ > 8 weeks
Q.11 How long is the mean duration of regular follow-up for a patient with LAS?
□ < 2 weeks □ 2–4 weeks □ 4–6 weeks □ 6–8 weeks □ > 8 weeks
3. Criteria for a return to sports (RTS) after conservative treatment for athletes with LAS
Q.12 What are your criteria for decision-making in relation to the RTS in patients with LAS who participate in sports activities?

## Results

**Fig. 1:**
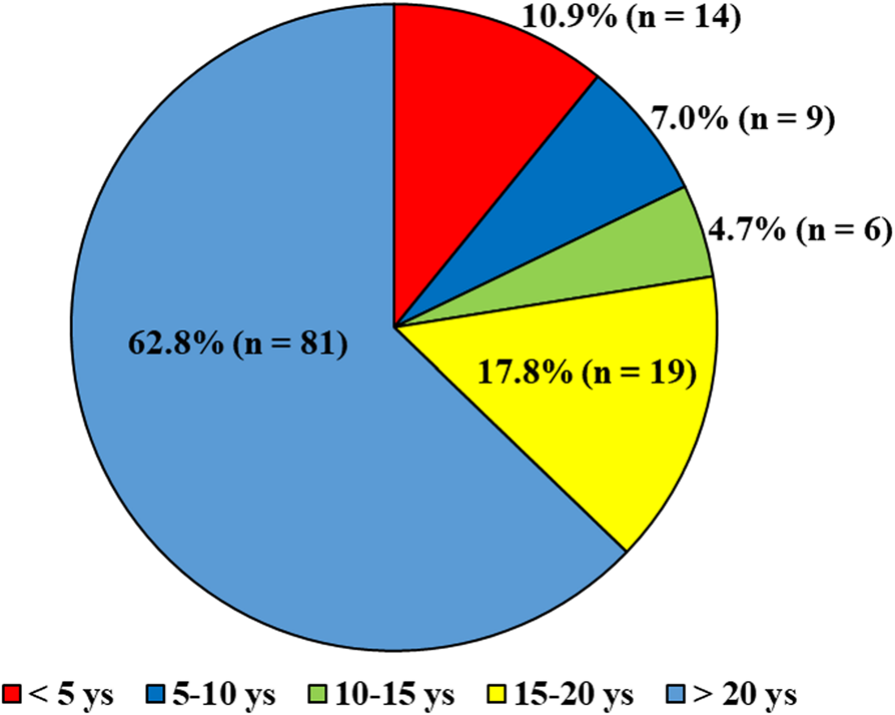
The experience of the orthopaedic doctors (n = 129). ys, years

A total of 156 invitations and questionnaires were sent out for the survey, and 129 completed the questionnaires in the study period. The response rate was 82.7%. Forty-eight private clinics (48 doctors) and 28 hospitals (81 doctors) were included. The experience as an orthopaedic surgeon was > 15 years in 99 (80.6%) (Fig. 1).

### The diagnosis of LAS (Question 1–4)

All doctors (100%, 129/129) answered that they performed plain radiography for the diagnosis of LAS (to exclude fractures). Only 4.7% (6/129) considered the OARs in the decision to perform plain radiography. Regarding stress radiography, 58.9% (76/129) answered that they did not perform stress radiography for the diagnosis of LAS. Ultrasonography was used by 46.5% (60/129) of the orthopaedic surgeons.

### Conservative treatment and rehabilitation for LAS (Question 5–11)

**Fig. 2:**
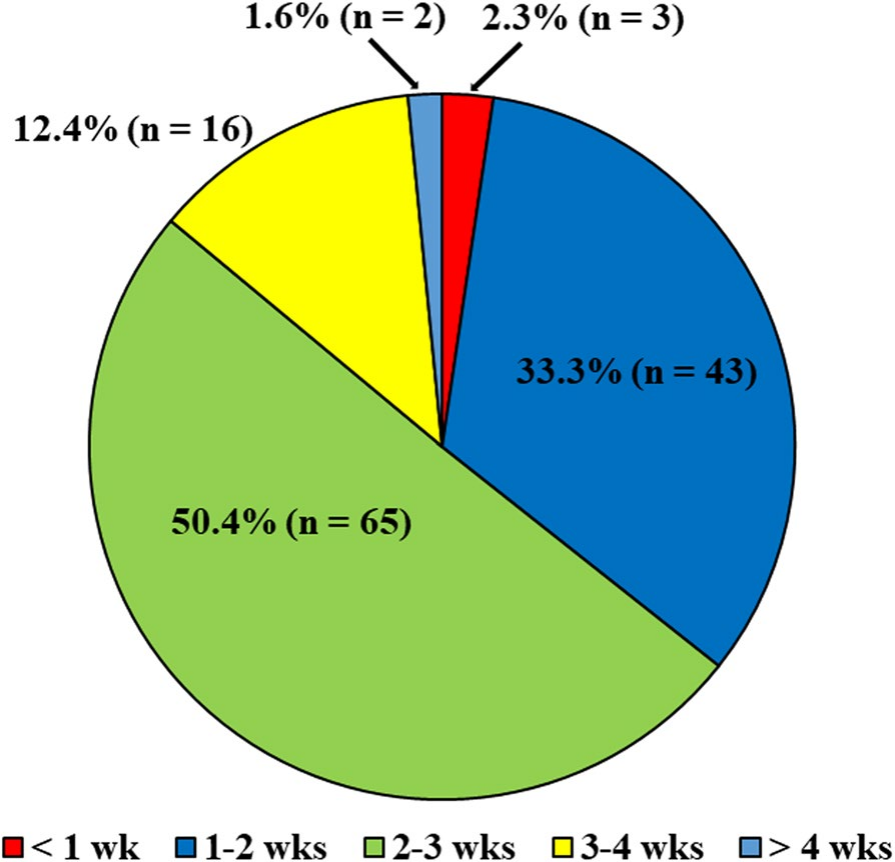
Duration of immobilization for patients with lateral ankle sprain. wk, week

Regarding immobilization for patients with LAS, 98.4% (127/129) immobilized the injured ankle for the treatment of LAS. A splint was applied for immobilization by 89.1% (115/127), and a cast was used by 6.2% (8/127). The duration of immobilization was reported to be < 1 week by 2.3% (3/129) of orthopaedic surgeons, and > 2 weeks by 64.4% (83/129) (Fig. 2). One hundred six respondents (83.5%) answered that they applied an ankle supporter after immobilization. With respect to whether or not to instruct a patient to avoid weight bearing on the injured foot, 23.3% (30/129) answered that they did not consider the duration of non-weight bearing (NWB) after the diagnosis of LAS. The duration for NWB was < 2 weeks in 52.7% (68/129), and > 2 weeks in 24.1% (31/129).

**Fig. 3:**
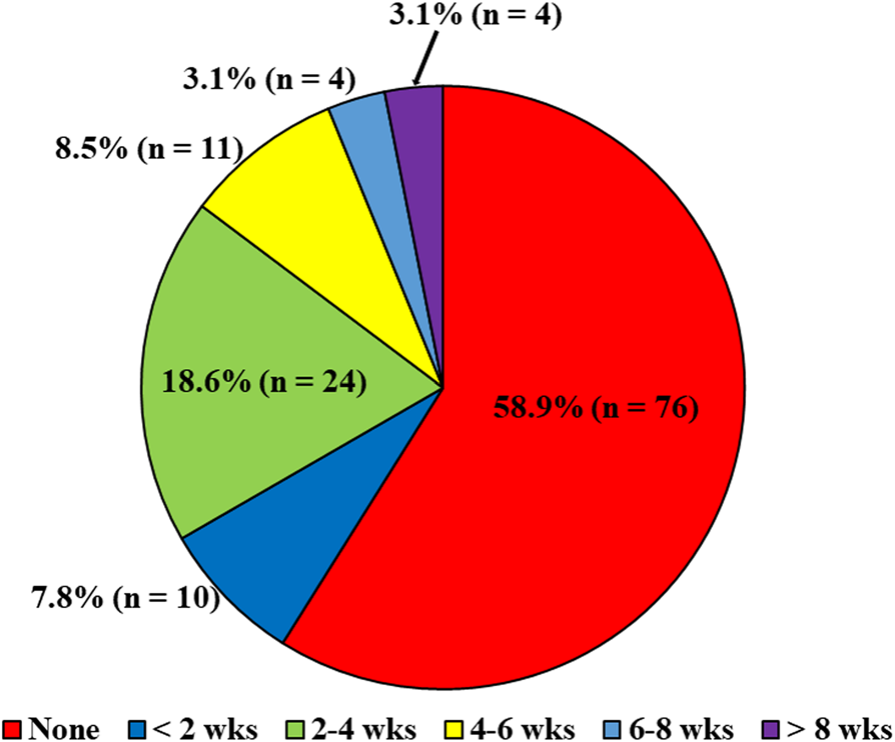
Prescription of rehabilitation for patients with lateral ankle sprain

Regarding the rehabilitation for LAS, 58.9% (76/129) of respondents answered that they did not order rehabilitation, while 85.3% (110/129) answered that they considered rehabilitation for < 4 weeks, and 3.1% (4/129) for > 8 weeks (Fig. 3). Twenty-one respondents (16.3%) answered that they did not instruct athletes with LAS to use an ankle supporter or taping during sports activities. Ninety-six of respondents (74.4%) answered that they instructed athletes with LAS to wear an ankle supporter during sports activities. The duration of regular follow-up was reported to be < 4 weeks by 35.8% (46/129), and > 8 weeks by 18.6% (24/129).

### The criteria for a RTS after conservative treatment for athletes with LAS (question 12)

**Fig. 4:**
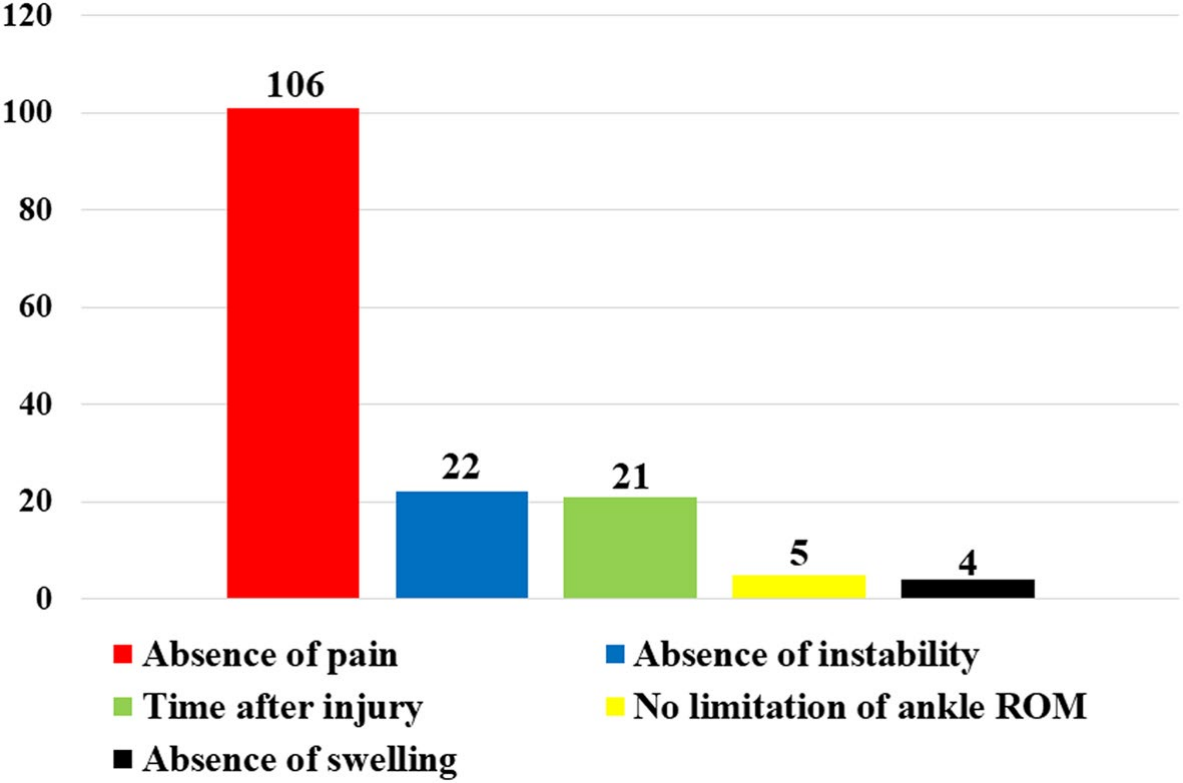
The top 5 factors used as criteria for a return to sports in athletes with lateral ankle sprain

As described in the Introduction, the question regarding the criteria for the RTS after conservative treatment for athletes with LAS was open-ended. The top 5 factors for the decision regarding the RTS were the absence of pain, the absence of instability, the time after LAS, no limitation of ankle ROM, and the absence of swelling (Fig. 4). Eighty-five (65.9%) of the respondents used only one of these 5 factors as the criterion for RTS; the absence of pain was the most frequently used criterion (45.7%, 59/129).

## Discussion

The main findings of the present study were as follows: (1) Only 4.7% of the orthopaedic surgeons considered the OARs in the decision to perform plain radiography for patients with suspected LAS; (2) Approximately 60% of the surgeons did not order rehabilitation for patients with LAS, and 85.3% reported that the duration of rehabilitation was < 4 weeks; (3) The orthopaedic surgeons did not use any objective criteria for the RTS decision after conservative treatment for LAS, and 65.9% used only one factor as a criterion for the RTS. Namely, most general orthopaedic surgeons did not provide the care recommended by the current CPGs to patients with LAS.

Ankle fracture should be excluded in the diagnosis of LAS. The OARs were introduced to rule out a fracture because the majority (> 80%) of individuals with LAS who underwent radiography did not have an ankle fracture [25]. A number of studies have shown the validity and usefulness of the OARs to avoid the unnecessary performance of plain radiography, especially in the emergency department [26–29]. The current CPG also documented that the OARs should be applied when an ankle fracture is suspected [10]. In this study, < 5% of orthopaedic surgeons considered the OARs in the examination of LAS, indicating that the OARs may not be popular in clinical practice by general orthopaedic surgeons in the management of LAS, and that plain radiography may be unnecessarily performed in some cases. Many studies were conducted in the emergency department setting or investigated treatment by general practitioners [15, 17, 26–28]. Therefore, general orthopaedic surgeons may pay limited attention to these studies or discussion on the OARs. However, Pires et al. found that a subjective analysis by orthopaedic surgeons to predict fractures had a higher specificity than the Ottawa ankle rules, suggesting that the clinical significance and usefulness of the Ottawa ankle rules might differ among emergent doctors and orthopaedic surgeons [30]. No studies, as far as we know, have yet resolved these issues. Further studies will be required to assess the difference in the recognition of OARs among emergency doctors, orthopaedic surgeons, and health providers. Papacostas et al. reported that the sensitivity of OARs was 100% when performed by orthopaedic residents or sports medicine doctors [27], suggesting the probability of a reduction in excessive radiation exposure and medical cost when orthopaedic physicians apply the OARs.

In the present study, 46.5% of the respondents reported using ultrasonography (US) to evaluate patients with LAS. Several authors have reported the validity of US in the assessment of LAS [31, 32]. Oae et al. reported that the accuracy of stress radiography and US in the diagnosis of anterior talofibular ligament injury were 67% and 91%, respectively, with the findings of arthroscopy as a reference [33]. Therefore, while the current CPGs do not mention the role of US in the evaluation of LAS [9, 10], the assessment of a fracture using US may result in an additional reduction of unnecessary radiography. More high-quality studies are needed to evaluate the cost-effectiveness of US, as well as the validity of US in the examination of acute LAS.

The current CPGs recommends that a rehabilitation program, including neuromuscular and proprioceptive exercise should be considered after LAS [3, 9, 10]. It has been demonstrated that exercise therapy reduces the rate of recurrence and functional instability [34, 35]. In the present study, < 60% of doctors prescribed rehabilitation for LAS. This rate may not be high, but was somewhat higher than that reported by Feger et al., who found that only 6.8% of patients with LAS received physical therapy within 30 days after the diagnosis of LAS in the US [13]. This may have been due to the fact that, in the present study, we did not evaluate what percentage of patients who were prescribed rehabilitation actually received rehabilitation. Our study population was also limited to orthopaedic surgeons. According to a systematic review, 45% of patients did not completely recover, with 25% complaining of pain and instability at 3 years after LAS [36]. In addition, considering that 74% of patients with LAS had not fully recovered after a mean follow-up period of 29 months [8], increasing the implementation of rehabilitation therapy by orthopedic surgeons will be necessary to improve the quality of care for LAS. Furthermore, the education of patients, their family, and athletic trainers and coaches—in the case of athletes, will be critical because the adherence to rehabilitation is directly related to the effectiveness of rehabilitation [37]. A poor understanding of the injury and the importance of rehabilitation therapy is a main factor in poor adherence [38]. McKay et al. reported that > 50% of athletes who suffered ankle injuries did not seek medical treatment [39]. Furthermore, it was reported that 64% of patients with CLAI did not seek medical treatment following an initial LAS [6]. These findings reflect a poor understanding of the severity of ankle sprain among patients with LAS.

In the present study, > 60% of the orthopaedic surgeons used only one of the 5 factors (absence of pain, absence of instability, time after LAS, no limitation of the ankle ROM, and absence of swelling) in the RTS decision after LAS. This finding suggests that athletes with LAS return to sports without insufficient evaluation in the clinical settings, which would result in a high risk of recurrent LAS and CLAI. A recent systematic review reported that there were no evidence-based criteria for the RTS decisions in athletes with LAS [19, 20]. The current CPGs recommended that early functional rehabilitation focused on muscle strength and response time, proprioception, and coordination be implemented to hasten RTS after LAS [9, 10]. Wikstrom et al. reported that a consensus was reached on the need to evaluate sports-specific movement in the decision of RTS after LAS; however, this statement was obtained by reviewing only low-level retrospective studies [20]. The lack of evidence regarding the criteria for RTS after conservative treatment has also been reported, not only in the LAS but also in knee ligament injuries [40, 41]. However, given the fact that almost half of LASs occur in athletes [1, 2], and that athletes with LAS are at a high-risk of recurrence [6, 39], high-quality studies are mandatory to construct definitive criteria for RTS after LAS.

Medina McKeon et al. reported that there was no difference in the RTS timeline between new and recurrent ankle sprains, and the most high-school athletes returned to sports within 1–3 days [22]. It was also reported that > 50% of athletes with LAS returned to sports in less than 1 week [36]. Considering that the ligament healing time that is at least more than 6 weeks [42], these findings suggest that the RTS after LAS is too early. In the present study, some surgeons used the time from injury as a criterion for the RTS decision; however, the time to RTS was not assessed in this study. It also remains unclear whether the time to RTS should be changed by the severity and a history (first-time or recurrent) of LAS. Malliaropoulos et al. reported that athletes with a low LAS grade (1 or 2) were at a higher risk of reinjury than those with grade 3 [43]. Further studies are needed to evaluate the relationship between the time to RTS and the recurrence rate after LAS.

There are several limitations in this study. First, we did not evaluate all of the issues described in the CPGs, such as the surgical therapy and the prescription of non-steroidal anti-inflammatory drugs. Second, most questions used in this study were close-ended in order to improve the response rate. Therefore, patient specific factors (age, activity level, severity of injury, etc.) were not completely considered. The present study aimed to assess the general clinical practice of orthopaedic surgeons for LAS, therefore, the results should be interpreted with caution. Third, the respondents to the questionnaire survey were all orthopedic surgeons in Miyazaki; therefore, the results in this study may not reflect the current clinical practice in other regions. As mentioned in the Introduction, the survey in the study region would be appropriate for evaluating the clinical practice by general orthopaedic surgeons in the management of LAS. The response rate of a survey of Canadian orthopedic surgeons to investigate the performance of microfracture surgery for knee chondral defects was 24.6% [44]; thus, the response rate of our study (82.7%) was quite high. In spite of these limitations, this study has clinical relevance, both in its emphasis of the need for further studies—especially in relation to evidence-based decision-making for the RTS—and in the suggestion that interventions to bridge the gap between researchers, clinicians and patients should be considered to improve the treatment of LAS.

## Conclusions

The present study reported the current management of LAS by general orthopaedic surgeons in Miyazaki, Japan. Most clinicians did not consider the OARs when performing plain radiography. Rehabilitation following LAS was performed for < 60% of patients with LAS, and no objective criteria for the RTS decision were used for athletes with LAS.

## Data Availability

All data are available from the corresponding author upon reasonable request.

## Abbreviations

LAS: Lateral ankle sprain
RTS: Return to sports
CPG: Clinical practice guideline
CLAI: Chronic lateral ankle instability
OAR: Ottawa ankle rule
ROM: Range of motion
US: Ultrasonography
